# Pre-treatment structural brain biomarkers predict response to repetitive transcranial magnetic stimulation in subjective tinnitus

**DOI:** 10.3389/fneur.2026.1808769

**Published:** 2026-06-24

**Authors:** Zhongling Ding, Bo Peng, Mengfang Gong, Hongxuan Qiu, Qian He, Xiaoting Zhu, Shiyu Kang, Xiaoliang Sheng, Jisheng Liu, Yakang Dai, Duo-Duo Tao

**Affiliations:** 1 Department of Otorhinolaryngology, The First Affiliated Hospital of Soochow University, Suzhou, China; 2 Suzhou Institute of Biomedical Engineering and Technology, Chinese Academy of Sciences, Suzhou, China; 3 Suzhou Medical College of Soochow University, Suzhou, China

**Keywords:** machine learning, precision medicine, predictive biomarkers, repetitive transcranial magnetic stimulation, structural magnetic resonance imaging, tinnitus

## Abstract

**Background:**

Variable efficacy of repetitive transcranial magnetic stimulation (rTMS) for tinnitus necessitates predictive biomarkers. Pre-treatment brain structural features may predict rTMS outcomes, given that tinnitus involves structural brain alterations and rTMS can induce neuroplastic changes.

**Objective:**

To identify pre-treatment brain structural biomarkers predictive of rTMS efficacy in subjective tinnitus.

**Methods:**

We prospectively enrolled 64 patients with subjective tinnitus and 18 healthy controls (HCs). Patients underwent a 2-week course of rTMS. High-resolution T1-weighted structural MRI (sMRI) was acquired, and 242 whole-brain morphometric features were extracted. Univariate analysis identified features differing between responders and non-responders, which subsequently were used to construct a machine learning model evaluated via 5-fold cross-validation and SHapley Additive exPlanations (SHAP) analysis. Feature significance was further interpreted through three-group comparisons among responders, non-responders, and HCs. Spearman correlation analyses were performed between structural features and clinical improvement scores (ΔVAS, ΔTHI) as well as baseline clinical measures.

**Results:**

Thirty-six patients (56.25%) were classified as responders. Ten regional features distinguished responders from non-responders, encompassing prefrontal, limbic, sensorimotor, and parietal networks. The predictive model (ExtraTreesGini_BAG_L1) achieved optimal performance (AUC = 0.85; accuracy = 0.77; precision = 0.71; recall = 0.97; F1-score = 0.82). SHAP analysis identified right pars triangularis of the inferior frontal gyrus (IFGtriang-R) gray matter volume (GMV) as the top predictor (positive influence). Three-group comparison revealed that IFGtriang-R GMV was significantly larger in responders (0.90 ± 0.08) than in both HCs (0.86 ± 0.06) and non-responders (0.86 ± 0.07), indicating a specific structural signature associated with positive treatment outcome. Spearman correlation analyses revealed that IFGtriang-R volume did not significantly correlate with ΔVAS or ΔTHI, and no structural feature showed a robust association with baseline clinical measures after Bonferroni correction.

**Conclusion:**

Responders were characterized by relative enlargement of the IFGtriang-R, suggesting a threshold effect of neuroplastic reserve conducive to rTMS efficacy. Pre-treatment sMRI assessment of this region may facilitate patient stratification for rTMS treatment, advancing precision neuromodulation for tinnitus.

## 1 Introduction

Subjective tinnitus, the perception of sound without an external source, affects approximately 10%−15% of the global population (1). Its clinical manifestations are highly variable, and its underlying pathophysiology remains incompletely understood, with considerable heterogeneity across patients (2). Currently, multidisciplinary comprehensive management represents the standard treatment model for tinnitus. However, the selection of therapeutic strategies remains largely empirical due to the lack of objective biomarkers capable of predicting treatment efficacy (2).

Repetitive transcranial magnetic stimulation (rTMS) is a promising neuromodulation technique that aims to restore dysfunctional brain network activity by non-invasively modulating cortical excitability, particularly within prefrontal and auditory cortical regions (3). The therapeutic effects of rTMS are thought to arise from the induction of long-term potentiation- or depression-like synaptic plasticity (4, 5). However, meta-analyses indicate substantial inter-individual variability, with only 40%−60% of patients achieving clinically meaningful improvement (6, 7). This unpredictability highlights the need for pre-treatment predictive biomarkers. In this context, there has been growing interest in identifying neuroimaging and clinical predictors of rTMS response in tinnitus and related conditions, with the overarching goal of moving from empirical, “trial-and-error” treatment selection toward individualized, biomarker-guided neuromodulation.

Converging evidence suggests that the pathophysiology of tinnitus has expanded beyond peripheral auditory impairment to encompass the reorganization of central brain networks (8), with neuroimaging studies elucidating the structural substrates of these network alterations (9–11). For example, a meta-analysis demonstrated that tinnitus patients exhibit increased gray matter volume (GMV) in the bilateral superior temporal gyri, right middle temporal gyrus, and angular gyrus, alongside reduced volume in the left medial superior frontal gyrus (12). Furthermore, machine learning approaches have identified discriminative brain regions including the right insula and left rostral middle frontal gyrus (MFG) (13). Rosemann et al. (14) found that tinnitus patients exhibit a significant increase in GMV in the right MFG. Notably, they observed that cortical thickness in the left MFG was positively correlated with cognitive ability in healthy controls (HCs), but negatively correlated in tinnitus patients. This paradoxical relationship suggests that in tinnitus, the prefrontal cortex may be chronically engaged in processing the tinnitus percept, thereby depleting cognitive control resources. Consequently, increased cortical thickness in this region is associated with poorer cognitive performance, reflecting a maladaptive compensatory response.

Beyond these cortical alterations, the limbic system is also implicated, as evidenced by the findings of Besteher et al. (15), who reported that reduced GMV in the left parahippocampal gyrus is associated with tinnitus and proposed that this structural alteration, being primarily predicted by the tinnitus diagnosis itself rather than psychological distress scores, may reflect an inherent feature of the tinnitus-related neural network. Collectively, these findings indicate that tinnitus is associated with widespread structural alterations in networks subserving cognitive control, emotional regulation, and auditory processing.

Several complementary approaches have been explored to predict rTMS or other treatment responses using neurophysiological, functional neuroimaging, or clinical variables (16). In major depressive disorder and post-stroke cognitive impairment, for example, EEG- or functional MRI (fMRI)-derived features combined with machine learning have achieved encouraging accuracies for forecasting neuromodulation or rehabilitation outcomes, demonstrating the feasibility of data-driven response prediction from brain activity patterns and clinical profiles (16–18).

In tinnitus, most attempts to identify rTMS responders have relied on demographic, audiological, or symptom-based factors, yet larger cohort studies have not identified robust demographic or clinical predictors of rTMS outcome, underscoring the limited utility of purely clinical models (19–22).

More recently, neuroimaging-based work in tinnitus has begun to use pre-treatment resting-state fMRI network signatures to guide the choice between different neuromodulatory or sound-based interventions, supporting the concept that pre-treatment brain network organization carries predictive information for treatment response, even though these studies have not focused on structural MRI morphometry or on prediction for a single standardized rTMS protocol (23–25).

Research indicates that rTMS can not only modulate neural activity but also induce structural reorganization within the brain. For instance, in patients with depression, rTMS targeting the left dorsolateral prefrontal cortex (DLPFC) can increase GMV in emotion regulation regions (e.g., anterior cingulate cortex and insula) (26). Volume increases in subcortical areas such as the thalamus have also been reported (27). In tinnitus specifically, Poeppl et al. (28) found that among chronic tinnitus patients treated with high-frequency left DLPFC and low-frequency temporal cortex rTMS, responders exhibited a reduction in GMV in specific brain regions including the left dorsolateral/ventrolateral prefrontal cortex, left operculo-insular cortex, and right inferior temporal cortex, whereas non-responders showed no such changes. These findings suggest that GMV reductions concurrent with symptom improvement may represent beneficial neuroplastic remodeling induced by rTMS. Although the precise mechanisms remain incompletely understood, these volumetric changes may be related to rTMS-mediated modulation of cortical excitability and the induction of long-term potentiation- or depression-like plasticity. However, this longitudinal work relies on group-level comparisons and does not provide individualized prediction of rTMS response based on pre-treatment neuroanatomy.

Machine learning (ML) provides a powerful framework for extracting predictive patterns from high-dimensional neuroimaging data. This approach has been successfully applied to predict treatment outcomes in various neuropsychiatric disorders, including major depressive disorder and post-stroke cognitive impairment (29–31). In tinnitus, ML has primarily been used for classification tasks based on neuroimaging features, such as distinguishing tinnitus patients from healthy controls (HCs) (13). However, a comprehensive ML model based on pre-treatment structural neuroanatomical features to predict individual response to rTMS has not yet been reported.

Notably, Poeppl's study (32) employed conventional group-level comparisons and was therefore unable to predict individual treatment outcomes from pre-treatment brain structure. This limitation underscores the inability of traditional statistical methods to capture individualized predictive patterns. Moreover, while most existing studies have focused primarily on GMV, complementary morphometric measures such as cortical surface area and thickness, which reflect distinct microstructural foundations, may provide a more comprehensive anatomical profile. Therefore, the present study employs machine learning to systematically integrate these multidimensional morphological features (GMV, surface area, thickness) to identify neuroimaging biomarkers predictive of rTMS response. Compared with EEG- or fMRI-based predictors that primarily index dynamic neural activity with relatively limited spatial or microstructural specificity, a structural MRI-based ML approach offers a complementary, anatomy-grounded perspective by leveraging stable markers of long-term network reorganization (33, 34). Structural MRI can be acquired in routine clinical practice and allows joint modeling of GMV, cortical thickness, and surface area across distributed prefrontal, limbic, auditory, and somatosensory systems, thereby capturing multidimensional “neuroplastic reserve” that may shape an individual's capacity to benefit from rTMS (34). Rather than replacing functional or electrophysiological biomarkers, structural MRI-based ML has the potential to augment and integrate with these existing methods within future multimodal predictive frameworks (35, 36). Therefore, a structural MRI-driven ML model specifically targeting rTMS response in tinnitus would be both complementary to prior EEG/fMRI and clinical approaches and highly relevant for precision neuromodulation (19, 35).

Despite the accumulating evidence for structural and functional brain alterations in tinnitus, and the initial attempts to use clinical, EEG, and functional MRI measures to forecast treatment outcomes, it remains unknown whether pre-treatment whole-brain structural MRI morphometry alone can reliably distinguish future rTMS responders from non-responders at the individual level. This constitutes a key gap in current knowledge that the present work seeks to address. We hypothesized that structural features encompassing prefrontal cognitive control, limbic emotion, auditory, and potentially interconnected somatosensory regions would distinguish rTMS treatment responders from non-responders. This study aims to: (1) identify pre-treatment structural MRI morphometric features that significantly differentiate rTMS responders from non-responders; and (2) construct an ML model using these features to predict individual treatment outcomes. By developing such a data-driven predictive model and revealing its key neuroanatomical drivers, this study aims to provide a preliminary foundation for personalized rTMS treatment planning in tinnitus.

## 2 Methods

### 2.1 Participant recruitment

**Figure 1:**
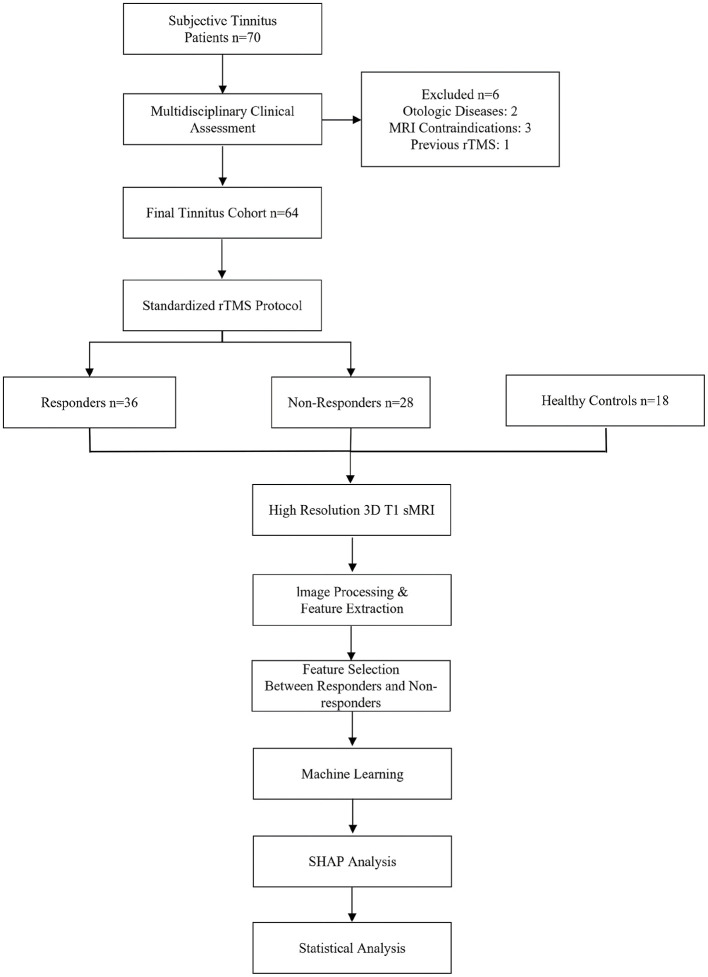
Workflow of study design.

Seventy subjective tinnitus patients and eighteen demographically matched healthy controls were prospectively recruited from the Department of Otorhinolaryngology at the First Affiliated Hospital of Soochow University between April and August 2025. Six patients were excluded due to otological disease history (*n* = 2), MRI contraindications (*n* = 3), or prior rTMS treatment (*n* = 1). Consequently, 64 patients were included in the final analysis (Figure 1). The protocol was approved by the Ethics Committee of the First Affiliated Hospital of Soochow University (Approval No.: 2025147). All participants provided written informed consent prior to enrollment.

Tinnitus patient inclusion criteria: (1) Adults (18–70 years); (2) Subjective tinnitus as primary complaint with duration ≥ 2 months; (3) Right-handedness; (4) Capacity to provide informed consent and complete assessments. Exclusion criteria included: (1) Objective tinnitus; (2) Meniere's disease, acoustic neuroma, or other otological pathologies; (3) Significant neurological or psychiatric disorders; (4) MRI contraindications; (5) Previous rTMS treatment.

HCs met the same exclusion criteria as patients, except for absence of tinnitus history. HCs were group-matched to the patient group on variables including age, sex, and binaural pure-tone average (PTA).

### 2.2 Multidisciplinary clinical assessment

Reflecting the multidisciplinary nature of modern tinnitus management, all patients underwent a comprehensive evaluation integrating expertise from otorhinolaryngology, audiology, neurology, and neuroradiology:

Audiological assessment: pure-tone audiometry was performed for each ear at test frequencies of 0.5, 1, 2, 4, and 8 kHz. The PTA was calculated as the mean of the thresholds at 0.5, 1, 2, and 4 kHz across both ears.

Tinnitus characterization: the Tinnitus Handicap Inventory (THI) was used to assess the functional, emotional, and catastrophic impacts of tinnitus (37). The Tinnitus Visual Analog Scale (VAS) was employed to evaluate subjective annoyance (38).

Neurological examination: a detailed neurological assessment was conducted by neurologists to exclude neurological conditions that could contribute to tinnitus or contraindicate rTMS treatment.

Structural MRI: as a core component of the pre-treatment evaluation, all participants underwent a high-resolution T1-weighted structural MRI scan.

### 2.3 Clinical characterization and response definition

Demographic and clinical characteristics were systematically documented, including age, gender, tinnitus laterality, duration, and pure-tone thresholds. Tinnitus severity was quantified using VAS (0–10) and THI (0–100) scores.

Treatment response was defined based on clinically meaningful thresholds assessed at the end of the 2-week rTMS treatment course. Patients were classified as responders if they met either of the following criteria after rTMS treatment (39): (1) A reduction of ≥ 1 point on the Tinnitus VAS score (40); (2) A reduction of ≥ 6 points in the total THI score (41). This dual-criterion approach captured both perceptual and functional dimensions of treatment success.

### 2.4 rTMS treatment protocol

rTMS was delivered using the Wuhan Yiruide CCY-I magnetic stimulator (39). Stimulation targets, the left dorsolateral prefrontal cortex (DLPFC) and the left temporal cortex (LTP), were localized using the F3 and T3 positions of the international 10–20 EEG system, respectively (42). Each patient wore a snug-fitting elastic cap, and the coordinates were marked by an experienced neurologist. The coil was placed tangential to the scalp with the handle pointing posteriorly. The stimulation intensity was individually calibrated to 110% of the resting motor threshold (RMT), which was defined as the minimum output required to elicit motor evoked potentials (MEPs) exceeding 50 μV in at least 5 out of 10 consecutive trials from the target muscle at rest (43). All participants completed a 10-session treatment protocol. Each session consisted of sequential stimulation: high-frequency rTMS was first applied to DLPFC (40 trains, 50 pulses per train, 25s intertrain interval, 20 Hz at 110% RMT), immediately followed by low-frequency rTMS to LTP (2,000 pulses at 1 Hz, 110% RMT) (28). This combined sequential protocol has been shown to produce a significantly greater number of treatment responders compared to sham stimulation and previous protocols (28). We adopted the identical protocol to enable direct comparison between our pre-treatment predictive findings and the post-treatment neuroplastic changes reported in that study, thereby obtaining a more comprehensive understanding of the rTMS mechanisms in tinnitus.

### 2.5 Multimodal MRI acquisition

High-resolution T1-weighted structural images were acquired using a GE Premier 3.0T scanner with a 3D T1-weighted magnetization-prepared rapid gradient-echo (MP-RAGE) sequence. All participants were provided with foam padding and earplugs to minimize head movement and noise exposure during scanning. Participants were instructed to remain still, keep their eyes closed, refrain from sleeping, and avoid engaging in specific cognitive tasks during the scan. The acquisition parameters were as follows: repetition time (TR) = 6.8 ms, echo time (TE) = 2.7 ms, flip angle (FA) = 8.0°, slice thickness = 1.0 mm (acquired without gap), isotropic voxel size = 1 × 1 × 1 mm3, acquisition matrix = 256 × 256, and field of view (FOV) = 256 × 256 mm^2^.

### 2.6 Image processing and feature extraction

T1-weighted MRI data were processed using Brainlab software (44) and comprised four main steps: (1) Image preprocessing: T1-weighted images were first resampled and reoriented into a standardized anatomical space. Intensity inhomogeneities were corrected using the N3 bias field correction algorithm. (2) Brain extraction: Non-brain tissues such as the skull, scalp, and dura were removed to isolate the brain structure. (3) Tissue segmentation and cortical reconstruction: the extracted brain images were segmented into gray matter, white matter, and cerebrospinal fluid. Based on the tissue segmentation, cortical surface reconstruction was performed to delineate the gray-white matter boundary and pial surface. (4) Brain parcellation: using the automated anatomical labeling (AAL) atlas, individual images were non-linearly registered to the template, yielding 90 brain regions and 76 cortical regions of interest (ROIs). The total intracranial volume (ICV) was calculated as the sum of gray matter, white matter, and cerebrospinal fluid volumes. Regional GMV values were then normalized by dividing by the individual's ICV to control for differences in overall brain size. All processing outputs were visually inspected for accuracy in segmentation and registration.

A comprehensive set of 242 morphometric features was extracted per subject. This comprised: normalized GMV for all 90 AAL-defined regions; cortical thickness and surface area for 76 cortical ROIs. Fourteen subcortical or deep gray matter structures (e.g., amygdala, caudate, thalamus) were excluded from thickness and surface area analyses, as these metrics are not reliably defined for such non-cortical regions. Raw data are presented in Mendeley Data (doi: 10.17632/cj3j8cwnc8.1).

### 2.7 Statistical analysis

Statistical analyses were performed using SPSS (version 29.0), with normality assessed by the Shapiro-Wilk test. Continuous variables are presented as mean ± standard deviation (SD) if normally distributed, or median (interquartile range) otherwise; categorical variables are presented as counts and compared using the Chi-square test. Between-group comparisons of baseline demographic and clinical characteristics employed independent samples *t*-tests or Mann–Whitney *U* tests for two-group comparisons.

To identify pre-treatment brain features that differed between future responders and non-responders, independent samples *t*-tests (for normally distributed variables) or Mann–Whitney *U* tests (for non-normally distributed variables) were applied to all 242 morphometric features. A two-tailed *p* < 0.05 was considered statistically significant. This univariate screening was performed for exploratory purposes. Therefore, the identified features should be interpreted as candidate biomarkers requiring further validation. For three-group comparisons, one-way analysis of variance (ANOVA) was used for normally distributed variables, followed by Bonferroni-corrected *post-hoc* pairwise comparisons if the overall test was significant (*p* < 0.05). For non-normally distributed variables, the Kruskal–Wallis *H* test was applied.

To explore the associations between key structural brain features and clinical improvement, as well as whether the selected structural features are independent of disease characteristics, we performed Spearman correlation analyses. Using ΔVAS (pre-treatment VAS—post-treatment VAS) and ΔTHI (pre-treatment THI—post-treatment THI) as continuous outcome measures, this study calculated their Spearman correlation coefficients with the key feature. Additionally, we calculated Spearman correlation coefficients between structural features showing intergroup differences and five baseline clinical measures (tinnitus duration, pre-treatment VAS, pre-treatment THI, PTA, and age). All analyses were based on data from all 64 patients, used two-sided tests, and considered *p* < 0.05 as statistically significant. To control for multiple comparisons, Bonferroni correction was applied with a corrected significance threshold of α = 0.001.

### 2.8 Construction and interpretation of a predictive model

The features that significantly differentiated responders from non-responders formed a parsimonious set for predictive modeling, aiming to evaluate the combined predictive utility of this neuroanatomical signature. A five-fold cross-validation framework was employed. In each iteration, four folds were used for training and the remaining fold for independent testing; this process was repeated five times so that each fold served as the test set once. Performance metrics were averaged across all test folds.

The following ensemble models were evaluated: ExtraTreesGini_BAG_L1 (45), WeightedEnsemble_L2 (46), NeuralNetFastAI_BAG_L1 (47), LightGBMLarge_BAG_L1 (48), and LightGBMXT_BAG_L1 (46). Model performance was evaluated using the area under the receiver operating characteristic curve (AUC), accuracy, precision, recall, and F1-score.

To interpret feature contributions within the best-performing model, SHapley Additive exPlanations (SHAP) values were calculated. SHAP quantifies each feature's marginal contribution to individual predictions, providing both a global importance ranking and a local explanation of how each feature value shifts the prediction toward responder or non-responder classification.

## 3 Results

### 3.1 Demographic and clinical characteristics of tinnitus patients and healthy controls

The tinnitus patient group (*n* = 64) and HCs (*n* = 18) were well-matched in age, gender, and bilateral pure-tone average (all *p* > 0.05). Details are provided in Table 1.

**Table 1:** Demographic and clinical characteristics of tinnitus patients and healthy controls.

Characteristic	Tinnitus (*n* = 64)	HCs (*n* = 18)	Statistics	*p*-value
Gender (m/f)	37/27	7/11	χ^2^ = 2.023	0.155
Age (years)	43.02 ± 11.80	37.94 ± 13.25	*t* = 1.568	0.121
PTA (dB HL)	18.87 ± 9.20	14.83 ± 5.91	*t* = 1.759	0.082
Tinnitus duration (months)	18 (6, 60)	NA	NA	NA
Tinnitus lateralization (right/left/bilateral)	18/11/35	NA	NA	NA
Pre-VAS	5 (4, 6.75)	NA	NA	NA
Pre-THI	34 (24, 50)	NA	NA	NA
Pre-THI-E	12 (8, 17.5)	NA	NA	NA
Pre-THI-F	12 (8, 22)	NA	NA	NA
Pre-THI-C	10 (6, 13.5)	NA	NA	NA

HC, health control; PTA, bilateral pure tone average hearing threshold across 500, 1,000, 2,000, and 4,000 Hz; Pre-VAS, pre-treatment tinnitus visual analog scale score; Pre-THI, pre-treatment tinnitus handicap inventory score; THI-E, emotional subscale of the THI; THI-F, functional subscale of the THI; THI-C, catastrophic subscale of the THI; NA, not applicable. Data are presented as mean ± standard deviation (SD) for normally distributed data, or median (first quartile, third quartile) [M (P25, P75)] for non-normally distributed data.

### 3.2 Structural brain differences between responders and non-responders to rTMS

After 2-week rTMS treatment, 36 patients (56.25%) were classified as responders and 28 as non-responders. No significant differences were found between responders and non-responders in any baseline clinical characteristics, including age, gender, hearing thresholds, tinnitus duration, laterality, or pre-treatment VAS/THI scores (all *p* > 0.05; Table 2). To identify pre-treatment brain structural features associated with treatment outcome, independent-samples *t*-tests were conducted on all 242 whole-brain morphometric features, revealing 10 regional features with statistically significant inter-group differences (all *p* < 0.05).

**Table 2:** Demographic and clinical characteristics of responders and non-responders.

Characteristic	Responders (*n* = 36)	Non-responders (*n* = 28)	Statistics	*p*-value
Gender (m/f)	22/14	15/13	χ^2^ = 0.367	0.545
Age (years)	42.44 ± 12.30	43.75 ± 11.32	*t* = −0.436	0.664
PTA (dB HL)	19.64 ± 9.85	17.88 ± 8.38	*t* = 0.755	0.453
8 kHz-R	25 (15, 45)	27.5 (16.25, 63.75)	*Z* = −1.393	0.164
8 kHz-L	22.5 (10, 42.50)	30 (15, 45)	*Z* = −1.252	0.211
Tinnitus duration (months)	18 (9, 48)	20 (5, 78)	*Z* = −0.312	0.755
Tinnitus lateralization (right/left/bilateral)	11/3/22	7/8/13	χ^2^ = 4.547	0.103
Pre-VAS	5 (4, 7)	5 (3.25,6)	*Z* = −0.370	0.711
Pre-THI	34 (24, 50)	31 (21, 50)	*Z* = −0.339	0.735
Pre-THI-E	10 (8, 16)	13(6.5,21.5)	*Z* = −0.795	0.427
Pre-THI-F	16 (8, 22)	9 (6, 22)	*Z* = −1.101	0.271
Pre-THI-C	10(6.5,14)	10 (6, 12)	*Z* = −0.513	0.608

Responders, rTMS treatment effective group; Non-responders, rTMS treatment ineffective group; PTA, bilateral pure tone average hearing threshold across 500, 1,000, 2,000, and 4,000 Hz; R, right; L, left; Pre-VAS, pre-treatment tinnitus visual analog scale score; Pre-THI, pre-treatment tinnitus handicap inventory score; THI-E, emotional subscale of the THI; THI-F, functional subscale of the THI; THI-C, catastrophic subscale of the THI. Data are presented as mean ± standard deviation (SD) for normally distributed data, or median (first quartile, third quartile) [*M* (P25, P75)] for non-normally distributed data.

Specifically, the GMV of the left middle frontal gyrus (MFG-L) was significantly greater in responders (0.68 ± 0.06) than in non-responders (0.64 ± 0.06; *t* = 2.40, *d* = 0.61, *p* = 0.019). Similarly, responders exhibited a significantly larger GMV in the right triangular part of the inferior frontal gyrus (IFGtriang-R; 0.90 ± 0.08) compared to non-responders (0.86 ± 0.07; *t* = 2.33, *d* = 0.59, *p* = 0.023). The cortical surface area of the left medial orbitofrontal cortex (ORBmed-L) was also larger in responders (1,008.95 ± 189.34 mm^2^) relative to non-responders (883.85 ± 172.51 mm^2^; *t* = 2.73, *d* = 0.69, *p* = 0.008). Furthermore, the responder group showed a significantly larger cortical surface area in the right postcentral gyrus (PoCG-R; 1,344.56 ± 160.16 mm^2^) than the non-responder group (1,257.72 ± 139.86 mm^2^; *t* = 2.27, *d* = 0.47, *p* = 0.027). The surface area of the right Heschl's gyrus (HES-R) was also greater in responders (1,214.66 ± 260.86 mm^2^) compared to non-responders (1,072.36 ± 301.88 mm^2^; *t* = 2.02, *d* = 0.51, *p* = 0.048). Additionally, the GMV of the left putamen (PUT-L) was significantly larger in responders (0.70 ± 0.04) than in non-responders (0.69 ± 0.03; *t* = 2.11, *d* = 0.53, *p* = 0.039).

Conversely, non-responders displayed a significantly larger GMV in the left medial superior frontal gyrus (SFGmed-L; 0.46 ± 0.03) than responders (0.43 ± 0.05; *t* = −2.26, *d* = −0.57, *p* = 0.028). The GMV of the left parahippocampal gyrus (PHG-L) was also higher in non-responders (1.37 ± 0.07) compared to responders (1.32 ± 0.08; *t* = −2.43, *d* = −0.61, *p* = 0.018). Similarly, the GMV of the right angular gyrus (ANG-R) in non-responders (0.54 ± 0.07) exceeded that in responders (0.50 ± 0.05; *t* = −2.28, *d* = −0.57, *p* = 0.026). Moreover, the cortical thickness of the left superior parietal gyrus (SPG-L) was significantly greater in non-responders (2.47 ± 0.15 mm) than in responders (2.40 ± 0.12 mm; *t* = −2.06, *d* = −0.52, *p* = 0.044).

**Figure 2:**
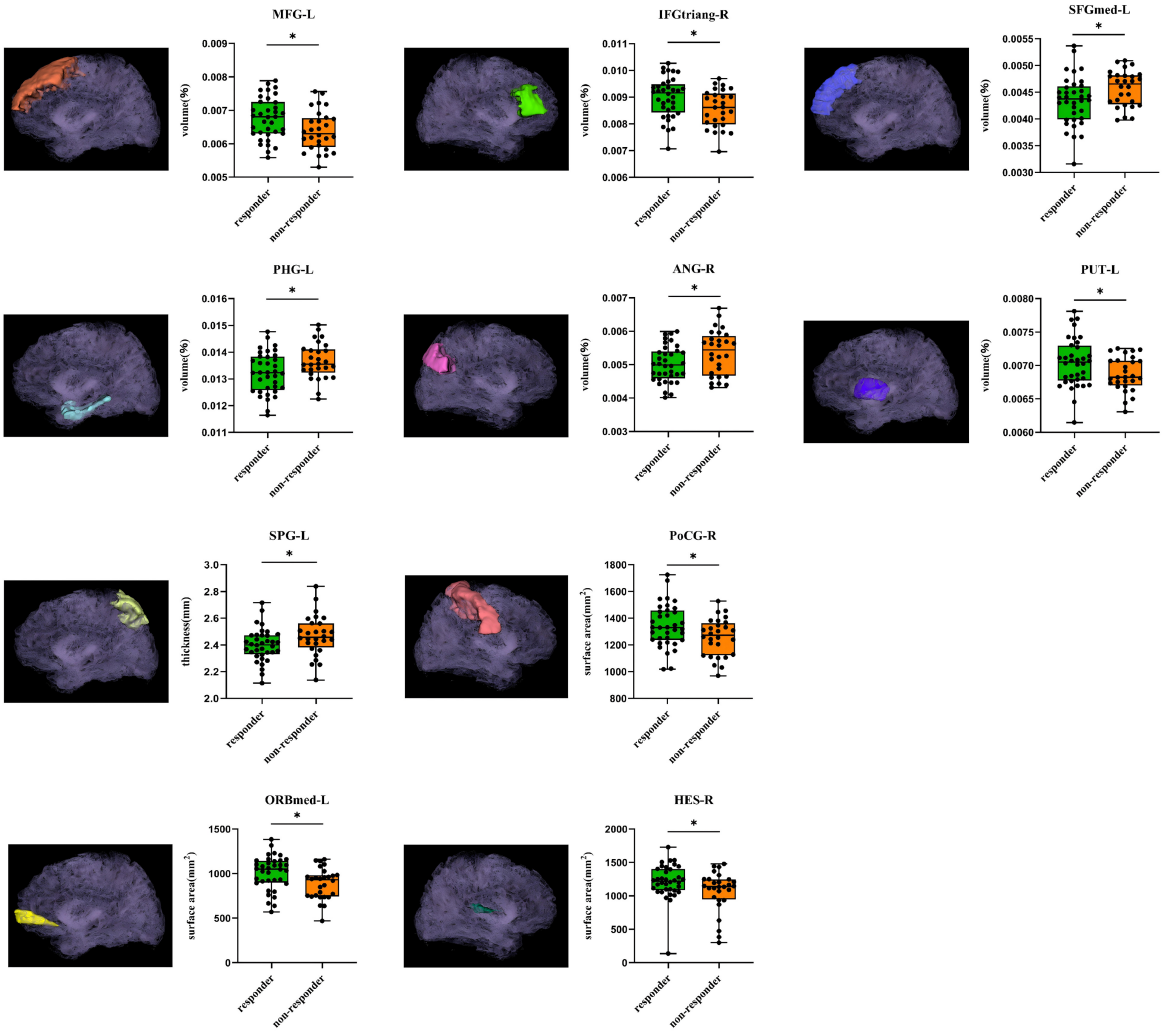
Structural differences between rTMS treatment responders and non-responders. Normalized volume was calculated as gray matter volume (GMV) divided by total intracranial volume (ICV), while thickness is expressed in mm and surface area in mm^2^. Responder, rTMS treatment effective group; Non-responder, rTMS treatment ineffective group; R, right; L, left; MFG, middle frontal gyrus; IFGtriang, inferior frontal gyrus, triangular part; SFGmed, superior frontal gyrus, medial part; PHG, parahippocampal gyrus; ANG, angular gyrus; PUT, putamen; SPG, superior parietal gyrus; PoCG, postcentral gyrus; ORBmed, medial orbital frontal cortex; HES, Heschl's gyrus. *p < 0.05. The lower boundary, midline, and upper boundary of the box represent the 25th percentile (Q1), the median (50th percentile), and the 75th percentile (Q3), respectively. The upper and lower whiskers extend to the maximum and minimum values of the data, respectively (i.e., the full range). Each scatter point in the figure represents the raw value of an individual sample.

These 10 discriminative regional features are distributed across networks involved in cognitive control, emotion regulation, sensory processing, and higher-order integration (Figure 2).

### 3.3 Predictive performance of the machine learning model based on discriminative features

**Figure 3:**
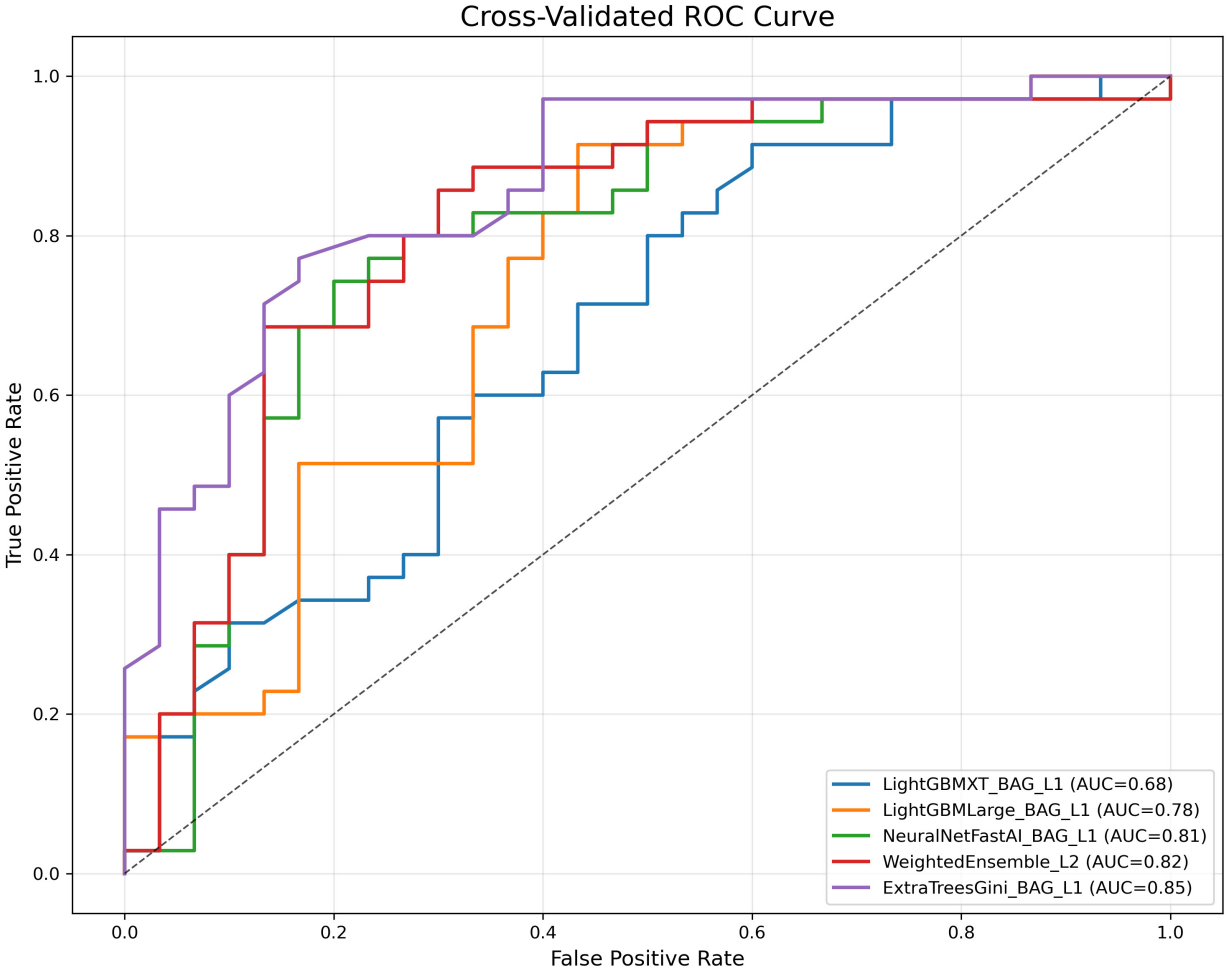
Receiver operating characteristic curves of the five ensemble machine learning models.

To evaluate the potential of these 10 discriminative features for predicting individual treatment outcomes, machine learning models were constructed and evaluated using five-fold cross-validation. The ExtraTreesGini_BAG_L1 model demonstrated the best performance, achieving an AUC of 0.85, accuracy of 0.77, precision of 0.71, recall of 0.97, and an F1-score of 0.82 (Table 3, Figure 3). Given the feature selection strategy, these metrics are exploratory estimates and require external validation.

**Table 3:** Performance comparison of different algorithm models (%).

Model	AUC	Accuracy	Precision	Recall	F1 score
ExtraTreesGini_BAG_L1	85.00	76.92	71.11	97.06	82.06
WeightedEnsemble_L2	82.38	73.85	68.95	94.29	79.53
NeuralNetFastAI_BAG_L1	81.43	69.23	66.95	85.71	74.48
LightGBMLarge_BAG_L1	77.62	64.62	60.79	97.14	74.70
LightGBMXT_BAG_L1	68.33	55.38	54.74	98.98	70.74

AUC, area under the curve.

**Figure 4:**
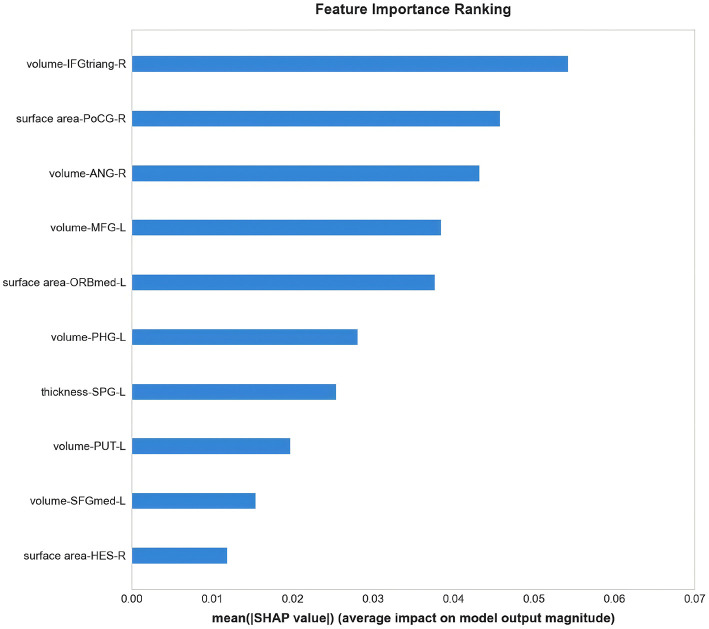
Feature importance ranking in the predictive model based on SHAP values. The plot displays the relative importance of the ten neuroanatomical features in the final predictive model, quantified and ranked in descending order by mean absolute SHAP value. Normalized volume was calculated as gray matter volume (GMV) divided by total intracranial volume (ICV), while thickness is expressed in mm and surface area in mm^2^.

**Figure 5:**
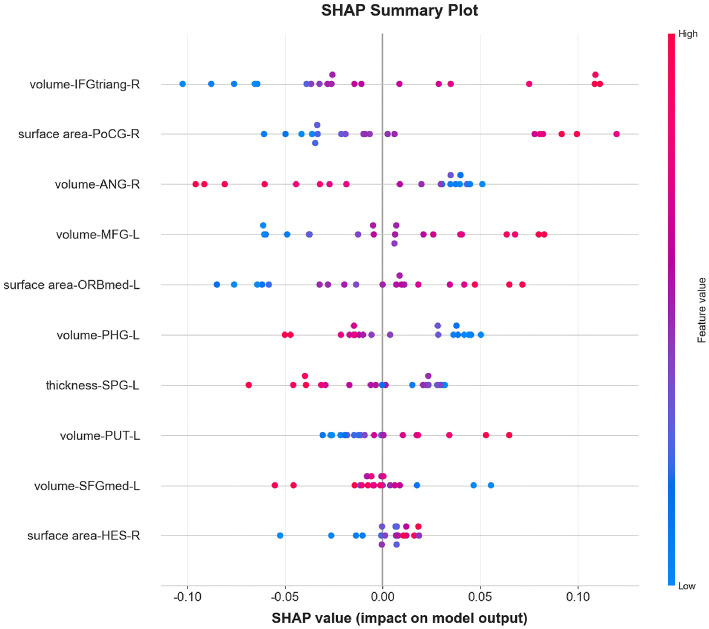
Association between feature values and model prediction direction (SHAP summary plot). This plot illustrates the relationship between each feature value and its impact on prediction (SHAP value). Each dot represents a sample, colored by feature value (red for high, blue for low), with horizontal position indicating impact on prediction (positive values push prediction toward “responder”). Features are ranked by importance. Normalized volume was calculated as gray matter volume (GMV) divided by total intracranial volume (ICV), while thickness is expressed in mm and surface area in mm^2^.

SHAP interpretability analysis revealed the contribution and direction of each feature's impact on the predictions. SHAP analysis identified GMV of IFGtriang-R as the most important predictor, with higher values associated with the prediction of treatment response. The remaining nine features, ranked in descending order of importance, were: surface area of PoCG-R (positive), volume of ANG-R (negative), volume of MFG-L (positive), surface area of ORBmed-L (positive), volume of PHG-L (negative), cortical thickness of SPG-L (negative), volume of PUT-L (positive), volume of SFGmed-L (negative), and surface area of HES-R (positive) (Figures 4, 5).

### 3.4 Comparative patterns of key structural features across three groups

To further analyze the neuroanatomical patterns of the key predictive features, one-way ANOVA was conducted to compare the three groups (responders, non-responders, and HCs) across the 10 aforementioned features (Table 4). The results showed statistically significant between-group differences in GMV of IFGtriang-R [*F*(2, 79) = 3.66, *p* = 0.030], the GMV of MFG-L [*F*(2, 79) = 3.40, *p* = 0.038], and the cortical surface area of ORBmed-L [*F*(2, 79) = 3.82, *p* = 0.026]. The remaining seven brain regions showed no statistically significant differences. For the three features that exhibited significant between-group differences, *post hoc* pairwise comparisons were performed using Bonferroni correction:

**Table 4:** Structural differences among rTMS treatment responders, non-responders, and healthy controls.

Function	Feature	Responders (*n* = 36)	Non-Responders (*n* = 28)	HCs (*n* = 18)	ω^2^	*F*	*p*-value	***p*** **-value**		
								Responder vs. non-Responder	Responder vs. HC	non-Responder vs. HC
Cognitive control	Volume-MFG-L	0.68 ± 0.06	0.64 ± 0.06	0.65 ± 0.06	0.055	3.397	0.038^*^	0.017^*^	0.080	0.732
	Volume-IFGtriang-R	0.90 ± 0.08	0.857 ± 0.07	0.859 ± 0.06	0.061	3.662	0.030^*^	0.017^*^	0.047^*^	0.916
	Volume-SFGmed-L	0.43 ± 0.05	0.46 ± 0.03	0.45 ± 0.06	0.027	2.139	0.125	NA	NA	NA
Emotion and reward regulation	Surface area-ORBmed-L	1,008.95 ± 189.34	883.85 ± 172.51	991.98 ± 202.45	0.064	3.821	0.026^*^	0.009^**^	0.754	0.059
	Volume-PHG-L	1.32 ± 0.08	1.37 ± 0.07	1.36 ± 0.08	0.046	2.990	0.056	NA	NA	NA
Sensory processing	Surface area-PoCG-R	1,344.56 ± 160.16	1,257.72 ± 139.86	1,293.51 ± 156.94	0.037	2.595	0.081	NA	NA	NA
	Surface area-HES-R	1,214.66 ± 260.86	1,072.36 ± 301.88	1,139.94 ± 248.53	0.027	2.153	0.123	NA	NA	NA
Higher-order integrative functions	Volume-ANG-R	0.50 ± 0.05	0.54 ± 0.07	0.53 ± 0.07	0.036	2.542	0.085	NA	NA	NA
	Thickness-SPG-L	2.40 ± 0.12	2.47 ± 0.15	2.44 ± 0.11	0.031	2.332	0.104	NA	NA	NA
	Volume-PUT-L	0.704 ± 0.04	0.69 ± 0.03	0.699 ± 0.04	0.023	1.969	0.146	NA	NA	NA

Normalized volume was calculated as the gray matter volume (GMV) divided by the total intracranial volume (ICV), while surface area is expressed in mm^2^. Responder, rTMS treatment effective group; Non-responder, rTMS treatment ineffective group; HC, health control; R, right; L, left; MFG, middle frontal gyrus; IFGtriang, inferior frontal gyrus (triangular); SFGmed, superior frontal gyrus (medial); PHG, parahippocampal gyrus; ANG, angular gyrus; PUT, putamen; SPG, superior parietal gyrus; PoCG, postcentral gyrus; ORBmed, orbitofrontal cortex (medial); HES, Heschl's gyrus; NA, not available. Comparisons among the three groups were performed using one-way ANOVA. If the ANOVA result was statistically significant (*p* < 0.05), *post hoc* pairwise comparisons were conducted using the Bonferroni correction. ^*^p < 0.05, ^**^p < 0.01. Due to the limited length of the article, only the statistically significant features among 242 are presented.

IFGtriang-R: The GMV in responders (0.90 ± 0.08) was significantly larger than that in non-responders (0.857 ± 0.07; *p* = 0.017) and HCs (0.859 ± 0.06; *p* = 0.047), indicating abnormal enlargement in responders relative to both groups. No significant difference was found between non-responders and HCs (*p* = 0.916).

MFG-L: the GMV in responders (0.68 ± 0.06) was significantly larger than that in non-responders (0.64 ± 0.06; *p* = 0.017). Although the comparison between responders and HCs (0.65 ± 0.06) showed a trend toward enlargement in responders, this difference did not reach statistical significance (*p* = 0.080). No significant difference was found between non-responders and HCs (*p* = 0.732).

ORBmed-L: the cortical surface area in responders (1,008.95 ± 189.34 mm^2^) was significantly larger than that in non-responders (883.85 ± 172.51 mm^2^; *p* = 0.009). Non-responders showed a trend toward reduced surface area compared to HCs (991.98 ± 202.45 mm^2^; *p* = 0.059). No significant difference was found between responders and HCs (*p* = 0.754).

In summary, among the three-group comparisons, only the GMV of IFGtriang-R showed a statistically significant difference between responders and HCs (*p* = 0.047), with responders exhibiting abnormal enlargement. Compared to HCs, MFG-L showed a trend toward enlargement in responders (*p* = 0.080), while ORBmed-L showed a trend toward reduced surface area in non-responders (*p* = 0.059).

### 3.5 Correlation analyses

#### 3.5.1 Correlation between structural features and baseline clinical measures

**Figure 6:**
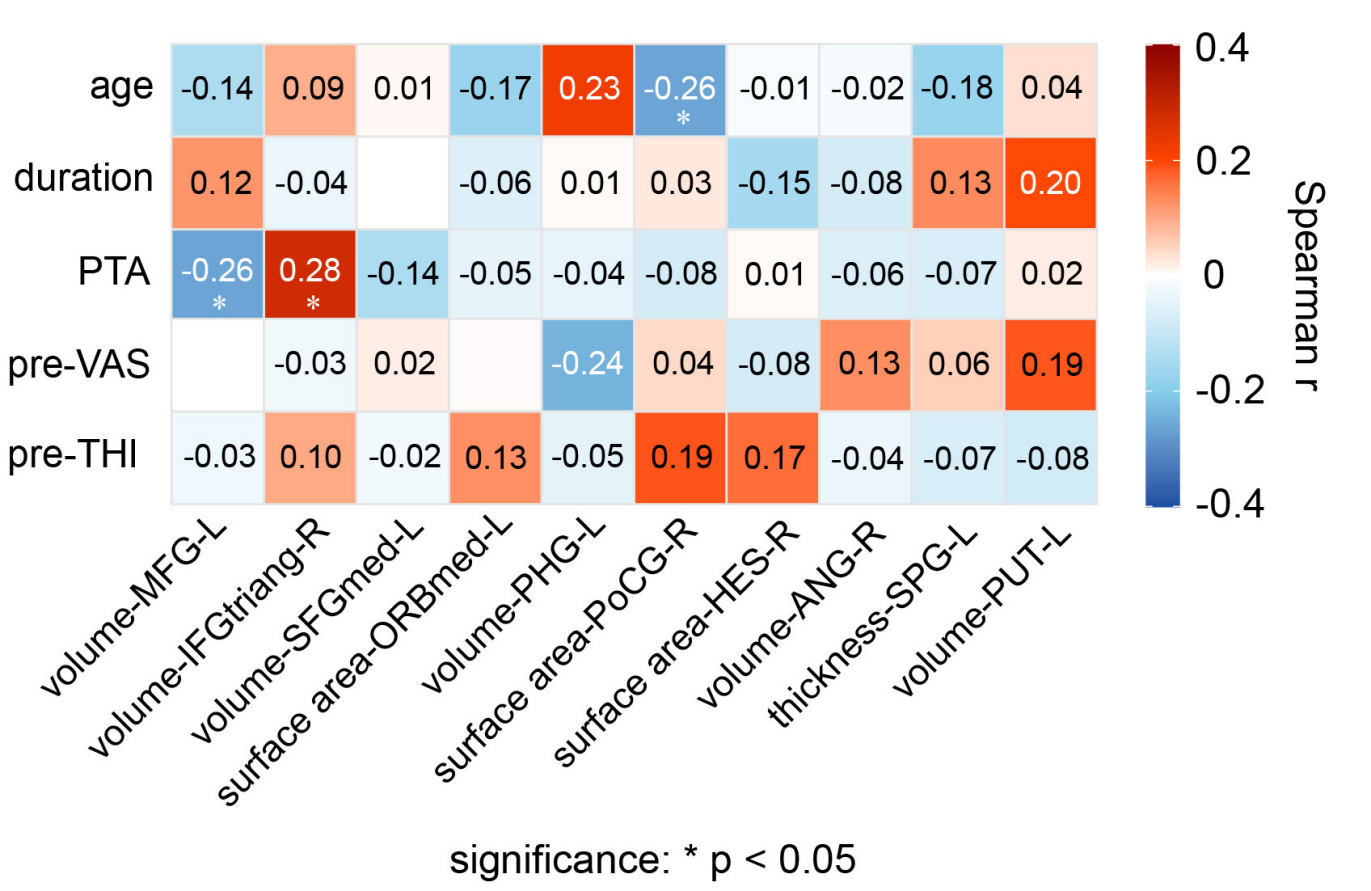
Spearman correlation matrix between ten structural features and clinical characteristics. Each cell shows the correlation coefficient (*r*). Red, positive correlation; blue, negative correlation. *p < 0.05. After Bonferroni correction (α = 0.001), no correlation remained significant.

Spearman correlation coefficients were calculated between the 10 structural features showing intergroup differences and five baseline clinical measures (tinnitus duration, pre-treatment VAS, pre-treatment THI, PTA, and age). Only three weak correlations were observed: MFG-L volume was significantly negatively correlated with bilateral PTA (*r* = −0.263, *p* = 0.036), IFGtriang-R volume was significantly positively correlated with bilateral PTA (*r* = 0.280, *p* = 0.025), and PoCG-R surface area was significantly negatively correlated with age (*r* = −0.261, *p* = 0.038). All other correlations were non-significant (*p* > 0.05) (Figure 6). After Bonferroni correction for multiple comparisons, none of the above *p*-values remained statistically significant. Therefore, these weak correlations should be interpreted as chance findings without robust statistical significance.

#### 3.5.2 Correlation between IFGtriang-R volume and degree of clinical improvement

IFGtriang-R was the top SHAP predictor and the only feature that significantly differed between responders and both non-responders and HCs (Table 4), suggesting it is the most specific structural biomarker. In all 64 patients, Spearman correlation analysis showed that the correlation coefficient between IFGtriang-R volume and ΔVAS was *r* = 0.212 (*p* = 0.093), and that with ΔTHI was *r* = 0.176 (*p* = 0.164); neither reached statistical significance (*p* > 0.05).

## 4 Discussion

This study identified 10 pre-treatment brain structural features that distinguished rTMS responders from non-responders through univariate group comparisons. These features were distributed across networks involved in prefrontal cognitive control, limbic emotion regulation, and sensory integration. A machine learning model (ExtraTreesGini_BAG_L1) constructed using these discriminative features demonstrated preliminary individual classification performance (AUC = 0.85). SHAP analysis further revealed that GMV of the right triangular inferior frontal gyrus (IFGtriang-R) emerged as the most influential positive predictor. A three-group comparison including healthy controls (HCs) revealed that responders exhibited significantly greater GMV in IFGtriang-R (0.90 ± 0.08) compared to both HCs (0.86 ± 0.06, *p* = 0.047) and non-responders (0.86 ± 0.07, *p* = 0.017), whereas no significant difference was observed between non-responders and HCs (*p* = 0.916). Collectively, these findings suggest that a predictive model based on pre-treatment structural MRI, with IFGtriang-R as its key driving feature, may identify patients likely to benefit from rTMS, thereby providing an objective neuroimaging foundation for precision neuromodulation therapy in tinnitus.

### 4.1 Structural basis of treatment response in responders

The findings indicate that patients who subsequently responded to rTMS exhibited significantly larger pre-treatment GMV in IFGtriang-R compared to non-responders. More importantly, comparison with HCs revealed that this volume enlargement in responders remained significantly elevated relative to the normal range (*p* = 0.047). We therefore regard IFGtriang-R enlargement as a structural signature associated with response rather than as definitive evidence of a qualitatively “healthier” brain state. We interpret this pattern not as unequivocal proof of a beneficial “reserve”, but as suggestive of prefrontal structural remodeling that may have emerged during the brain's long-term effort to cope with the chronic perceptual and cognitive demands of tinnitus. Consistent with cognitive load theory, tinnitus persistently engages perceptual resources within the auditory pathway while concurrently requiring the prefrontal cortex to mobilize cognitive resources for attentional control (49). This sustained, high-demand recruitment of cognitive resources may drive adaptive structural reorganization in the involved prefrontal cortices, although cross-sectional MRI cannot by itself establish whether such reorganization is adaptive or maladaptive.

Importantly, structural alterations in chronic sensory and pain conditions are often interpreted as maladaptive. Chronic pain, for example, is associated with consistent GMV reductions and even neurodegenerative-like changes in prefrontal, insular, thalamic, and cingulate regions, which have been linked to symptom severity and disease duration (50). From this perspective, our data do not justify the claim that IFGtriang-R enlargement is necessarily a “plasticity reserve” or advanced “adaptive remodeling”; it could also represent a late stage of chronic reorganization with unclear functional valence.

At the same time, several lines of evidence suggest that greater or increasing prefrontal/IFG GMV can, in some contexts, be associated with preserved functional potential or treatment-related improvement. Training studies have shown that increases in right IFG GMV parallel improvements in inhibitory control, leading to the conclusion that “the rIFG GMV is a determinant of IC proficiency” (51). In chronic pain, an 11-week CBT program resulted in increased GM in dorsolateral and ventrolateral prefrontal cortices that exceeded levels in healthy controls; these changes were interpreted as reflecting greater top-down control and cognitive reappraisal of pain rather than progression of pathology (52). Likewise, in chronic whiplash-associated disorders, right DLPFC GMV increased toward normalization after treatment, suggesting at least partial reversal of pain-related structural adaptations (53). In major depressive disorder, meta-analytic evidence indicates that responders tend to show relatively higher GMV in frontal and cingulate regions compared with non-responders and, in some regions, even compared with healthy controls, possibly reflecting treatment-related plasticity or preserved structural capacity that supports remission (54). Furthermore, in cocaine addiction, longitudinal increases in IFG GMV during abstinence have been associated with improved decision making and cognitive flexibility, interpreted as cortical recovery rather than further deterioration (55).

Against this background, our tentative interpretation is that relative IFGtriang-R enlargement in tinnitus responders may index a preserved or augmented capacity within the prefrontal inhibition/control network that can be recruited by high-frequency DLPFC rTMS, rather than solely reflecting disease burden. However, we explicitly acknowledge that this “neuroplastic reserve” framework is speculative, and that the same structural phenotype could also represent a more advanced stage of chronic reorganization in a subset of patients. Disentangling these possibilities will require longitudinal and multimodal studies that track both GMV trajectories and functional network changes in relation to clinical outcomes.

Our findings present an informative contrast to those reported by Poeppl et al. (28). In their longitudinal study, treatment responders exhibited rTMS-induced GMV reductions in prefrontal and temporal regions, whereas our cross-sectional data identify pre-treatment IFGtriang-R enlargement as a predictor of response. Integrating these observations, we cautiously propose a theoretical baseline-reserve vs. induced-consequence model, in which pre-existing prefrontal enlargement may represent a structural predisposition that can be further remodeled by rTMS. However, in the absence of longitudinal imaging in the present cohort, this baseline reserve vs. induced consequence framework should be regarded as a speculative conceptual model rather than a verified mechanism.

We further propose that patients exhibiting abnormal enlargement within the prefrontal cognitive control network prior to treatment may possess a richer, though not necessarily purely beneficial, substrate for plastic change. When receiving high-frequency rTMS targeting the DLPFC, these structurally advantaged brain regions may demonstrate enhanced excitatory responsiveness and more effectively translate stimulation into top-down inhibitory control signals (56, 57), thereby enhancing regulation of aberrant activity in downstream limbic and auditory cortices (10, 58, 59). Notably, beyond the significant finding in IFGtriang-R, responders showed a trend toward larger GMV in the left middle frontal gyrus (MFG-L) compared to HCs, while non-responders exhibited a trend toward reduced surface area in the left medial orbital frontal cortex (ORBmed-L). These observations suggest that broader prefrontal-limbic system features may contribute to differentiating treatment outcomes (10, 60, 61). Future longitudinal and multimodal work is needed to clarify whether such prefrontal enlargements primarily reflect maladaptive hypertrophy, beneficial reserve, or a mixture of both, and how these structural states evolve in response to rTMS.

### 4.2 Relationship between structural features and baseline clinical measures

The study found that none of the 10 brain morphometric features showed a significant and robust correlation with core disease indicators such as tinnitus duration or pre-treatment severity (VAS, THI). Only three weak correlations were observed (MFG-L volume and IFGtriang-R volume with PTA; PoCG-R surface area with age), and none remained significant after correction for multiple comparisons. More importantly, responders and non-responders did not differ significantly in any baseline clinical variables (Table 2). These findings suggest that the brain morphometric features are not simple proxies for disease severity or duration, but rather relatively independent predictors of treatment response. The absence of robust correlations with baseline THI/VAS or tinnitus duration suggests that these features index relatively enduring, trait-like neuroplastic states that are only partially coupled to current symptom burden, rather than linear readouts of severity or chronicity.

### 4.3 Integrative value of the machine learning model

The machine learning model developed in this study (ExtraTreesGini_BAG_L1) integrates ten key structural features from prefrontal, limbic, sensorimotor, and parietal networks to help predict individual responses to rTMS treatment. The model's performance metrics suggest potential clinical relevance: an AUC of 0.85, which exceeds the threshold for good discriminative ability (>0.80), indicates preliminary capacity to distinguish future responders from non-responders (62, 63); a precision of 0.71 suggests that approximately 70% of patients classified as “likely responders” subsequently exhibited symptom improvement. Relative to the overall response rate of 0.56 in the present study, this may represent a meaningful shift from empirical “trial-and-error” approaches toward data-driven patient selection strategies; a recall of 0.97 indicates a minimal false-negative rate (0.03) for true responders; and an overall accuracy of 0.77 suggests that the model provides correct outcome predictions for over three-quarters of patients. These findings provide a preliminary neuroimaging basis that could, after further validation, aid in clinically identifying tinnitus patients likely to benefit from rTMS therapy.

### 4.4 Clinical implications and translational prospects

Our findings may carry dual implications for clinical translation after appropriate validation. First, at the practical level, the prediction model developed using routine T1-weighted MRI may offer a feasible approach for objective patient stratification prior to rTMS treatment. This could, after prospective validation, provide a complementary data-driven perspective to current empirical decision-making, which may in the future contribute to the precision of multidisciplinary tinnitus care. Second, at mechanistic level, the identification of IFGtriang-R as a key predictive brain region deepens our understanding of the neural basis underlying rTMS efficacy. These findings suggest that the pre-existing structural state of the prefrontal cognitive control network represents an important factor determining sensitivity to neuromodulatory interventions, providing novel perspectives for future optimization of stimulation parameters and target selection.

### 4.5 Limitations and future directions

This study has several limitations. First, while our findings support within-site generalizability under these constraints, they should be considered preliminary until replicated in larger or multi-site cohorts. Second, univariate feature selection was performed without correction for multiple comparisons, and both feature selection and model evaluation were conducted on the same dataset, which may introduce optimistic bias. Thus, these findings should be interpreted as exploratory and require confirmation via more rigorous procedures, such as nested cross-validation, independent external validation cohorts, or multivariate feature selection strategies that account for multiple comparisons. Third, treatment response was assessed only at the end of the 2-week course without long-term follow-up; whether these structural features predict sustained efficacy (e.g., at 3 or 6 months) remains to be further investigated.

Fourth, the three-group comparison revealed that IFGtriang-R GMV was significantly larger in responders than in both HCs and non-responders, but the responder-HC contrast was only marginally significant (p = 0.047) and based on a relatively small responder subgroup (*n* = 36). This putative “neuroanatomical subgroup” effect should therefore be regarded as statistically fragile and hypothesis-generating rather than definitive evidence that responders constitute a distinct structural subtype compared to the healthy population. Future studies with substantially larger samples and independent external cohorts will be essential to determine whether the observed IFGtriang-R enlargement in responders is reproducible and robust across centers and imaging platforms.

Fifth, our prediction model was intentionally restricted to pre-treatment structural MRI morphometry. No systematic EEG, MEP/rMT, or blood-based biomarker data were collected in this cohort, and such modalities could not be integrated as additional predictors. As a result, the current model captures only one facet of the complex pathophysiology of tinnitus and rTMS treatment effects. Although structural MRI offers a stable and clinically accessible window into long-term network reorganization, future multimodal studies are needed to examine whether combining sMRI with validated neurophysiological or biochemical markers can further improve predictive performance, while carefully balancing model complexity, generalizability, and feasibility in real-world clinical workflows.

Sixth, using a tinnitus duration of ≥2 months as an inclusion criterion may introduce pathophysiological heterogeneity, although analyses showed no significant association between disease duration and the core results. Seventh, we used 10–20 EEG-based (F3/T3) rather than neuronavigated targeting. This approach involves typical deviations of 10–20 mm (64, 65), which may introduce noise to treatment response classification. Nevertheless, depression rTMS studies show that different scalp-based methods achieve similar clinical outcomes despite such deviations (66). Any unsystematic mis-targeting is therefore more likely to weaken than to artificially create our observed structure-response associations. Eighth, the intense scanner noise may temporarily aggravate tinnitus. Future studies could employ noise-free or low-noise techniques such as functional near-infrared spectroscopy (fNIRS), which has been combined with machine learning to predict treatment response in tinnitus (67–69). Integrating structural MRI with silent fNIRS may enable a more comprehensive and clinically applicable predictive model.

## 5 Conclusion

This study identifies a neuroanatomical profile based on pre-treatment structural MRI that could be used to predict therapeutic response to rTMS in patients with tinnitus. Among these features, increased GMV in IFGtriang-R serves as a core predictive biomarker, potentially representing a reserve of neural plasticity conducive to neuromodulation efficacy. However, given the exploratory nature of this study, the model should currently be viewed as a hypothesis-generating tool. Prospective validation in blinded, multi-center trials is required before any clinical application can be considered.

## Data Availability

The datasets presented in this study can be found in online repositories. The names of the repository/repositories and accession number(s) can be found below: Mendeley Data (https://doi.org/10.17632/cj3j8cwnc8.1).
